# Right-Sided Empyema Thoracis Mimicking Pulmonary Tuberculosis Secondary to a Complicated Hepatic Cyst: A Diagnostic Pitfall

**DOI:** 10.7759/cureus.108362

**Published:** 2026-05-06

**Authors:** Fatema Abdulkarim, Aaliyah Kheruwala, Wafa Erabia, Bassam Mahboub

**Affiliations:** 1 Pulmonology, Rashid Hospital, Dubai, ARE; 2 Internal Medicine, Rashid Hospital, Dubai, ARE

**Keywords:** empyema thoracis, hepatic cyst, pleural effusion, transdiaphragmatic spread, tuberculosis mimic

## Abstract

Pleural empyema commonly presents with constitutional and respiratory symptoms that may closely mimic pulmonary tuberculosis (TB), particularly in patients from TB-endemic regions. We report a diagnostically challenging case of a 42-year-old man admitted with chronic cough, weight loss, night sweats, and a massive right-sided pleural effusion, initially managed under airborne isolation as suspected pulmonary TB. Comprehensive pleural fluid analysis demonstrated an advanced bacterial empyema, while molecular and microbiological testing excluded TB. Thoracoabdominal computed tomography (CT) unexpectedly identified a large (12.5 x 9 cm), multiloculated cystic lesion in the right hepatic lobe exhibiting mass effect and transdiaphragmatic extension into the pleural space. Subsequent ultrasound-guided liver aspiration confirmed a pyogenic liver abscess (PLA), with fluid cultures definitively growing *Klebsiella pneumoniae*. This case highlights an uncommon extra-thoracic source of pleural infection (a "silent" *Klebsiella* liver abscess) that completely masqueraded as pulmonary TB due to the overshadowing respiratory symptoms. It underscores the critical importance of evaluating the subdiaphragmatic space via extended thoracoabdominal imaging in cases of unexplained right-sided empyema. Early recognition of invasive *Klebsiella* syndromes is essential to prevent diagnostic anchoring, ensure targeted antimicrobial therapy, and achieve simultaneous dual-compartment source control.

## Introduction

Pleural empyema remains a significant cause of morbidity worldwide, particularly among patients presenting with prolonged constitutional symptoms and radiological evidence of pleural space disease [1,2]. Despite advances in antimicrobial therapy and imaging, empyema continues to pose diagnostic and therapeutic challenges, especially when its presentation overlaps with endemic infectious diseases such as tuberculosis (TB) [3,4]. In TB-endemic regions and among high-risk populations, pleural effusion accompanied by weight loss, night sweats, and anemia is frequently presumed to be tuberculous in origin, often prompting early isolation and empiric diagnostic pathways [3,5].

However, non-tuberculous causes of exudative pleural effusion and empyema are increasingly recognized, particularly in the context of complex intra-thoracic or intra-abdominal inflammatory processes. Bacterial empyema is biochemically characterized by elevated lactate dehydrogenase (LDH) levels, markedly reduced pleural glucose, and neutrophil predominance, reflecting advanced pleural infection and fibrinopurulent organization [4]. When untreated or diagnosed late, empyema may progress to loculation, pleural thickening, and lung entrapment, necessitating invasive drainage or surgical intervention [6,7].

An under-recognized mechanism in the pathogenesis of pleural empyema is transdiaphragmatic spread of infection from subdiaphragmatic sources, including hepatic, subphrenic, and intra-abdominal collections. The close anatomical relationship between the liver, diaphragm, and pleural space allows inflammatory or infectious processes to extend across tissue planes, particularly when chronic inflammation weakens diaphragmatic barriers [8]. Hepatic cystic lesions, while commonly benign and incidental, may rarely become complicated by infection, hemorrhage, or surrounding inflammatory edema, with potential secondary involvement of adjacent thoracic structures [9,10].

Complicated hepatic cysts presenting primarily with pulmonary or pleural manifestations are uncommon and frequently misdiagnosed, especially when respiratory symptoms dominate the clinical picture. In such cases, chest imaging may reveal empyema or pleural effusion, while the underlying abdominal pathology remains occult unless cross-sectional imaging is performed [8]. This diagnostic delay may contribute to prolonged illness, inappropriate antimicrobial strategies, and unnecessary isolation for presumed pulmonary TB.

We report the case of a middle-aged man admitted with constitutional and respiratory symptoms, initially admitted as suspected pulmonary TB, who was ultimately diagnosed with right-sided empyema associated with a complicated hepatic cyst exhibiting surrounding inflammatory edema. This case highlights the importance of comprehensive imaging, careful pleural fluid interpretation, and maintaining a broad differential diagnosis in TB-suspected patients. It also underscores the clinical relevance of hepatic-pleural inflammatory interactions, an area that remains sparsely reported in pulmonology literature.

## Case presentation

Patient information and presenting symptoms

A 42-year-old Bangladeshi male (weight: 51 kg, height: 160 cm), working as a driver and residing in a crowded accommodation, was admitted with a one-month history of persistent dry cough, intermittent night sweats, low-grade fever, progressive fatigue, and an unintentional weight loss of approximately 8 kg. He denied hemoptysis, chest pain, or gastrointestinal symptoms. A history of travel to Bangladesh six months prior, coupled with his demographic background and constitutional symptoms, raised a high initial clinical suspicion for pulmonary TB.

Clinical examination and initial laboratory findings

On admission, vital signs revealed tachycardia (HR 132 bpm) and elevated blood pressure (151/84 mmHg), with a borderline temperature of 37.6 °C and an oxygen saturation of 100% on room air. Physical examination was notable for markedly reduced air entry over the right hemithorax. Initial laboratory investigations demonstrated anemia (Hb 7.7 g/dL, MCV 71.4 fL) and elevated inflammatory markers, including a C-reactive protein (CRP) of 74.7 mg/L and an erythrocyte sedimentation rate (ESR) of 58 mm/hr. Procalcitonin was 0.31 ng/mL, and HbA1c was normal at 4.8%. Iron studies were suggestive of anemia of chronic disease with co-existing iron deficiency (serum iron 12 µg/dL, transferrin saturation 7%, and paradoxically elevated ferritin 1,175 ng/mL). Liver function testing revealed hypoalbuminemia (2.6 g/dL), elevated alkaline phosphatase (227 U/L), hyperglobulinemia (4.5 g/dL), and total protein of 7.1 g/dL, with preserved ALT (27 U/L) and total bilirubin (0.70 mg/dL). Comprehensive viral screening (human immunodeficiency virus, HIV; hepatitis B/C; severe acute respiratory syndrome coronavirus 2, SARS-CoV-2; influenza; respiratory syncytial virus, RSV) and blood cultures were negative.

Initial imaging and thoracentesis

**Figure 1:**
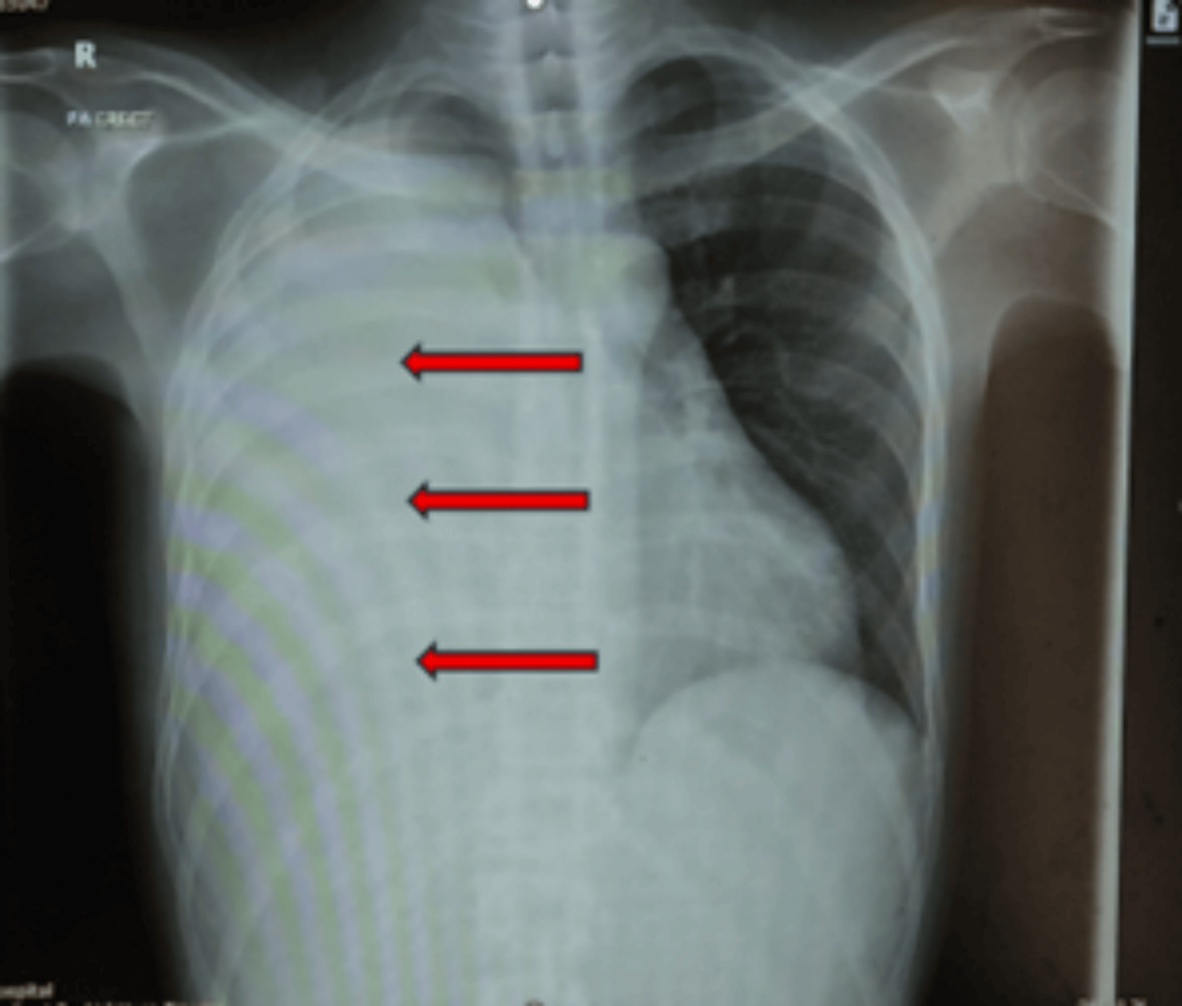
Initial posteroanterior (PA) erect chest X-ray demonstrating near-complete opacification of the right hemithorax, consistent with a massive pleural effusion

A posteroanterior erect chest radiograph demonstrated near-complete homogeneous opacification of the right hemithorax with contralateral mediastinal shift, consistent with a massive pleural effusion, as shown in Figure 1.

Diagnostic and therapeutic thoracentesis was performed, which yielded an initial 400 mL of yellow, turbid fluid. Pleural fluid analysis was characteristic of a neutrophilic inflammatory exudate, revealing a total nucleated cell count of 18,185 cells/mm³ (95% neutrophils, 5% monocytes), markedly elevated LDH (4,852 U/L), total protein of 5.3 g/dL, and critically low glucose (<2 mg/dL). Pleural fluid cytology was negative for malignant cells but consistent with empyema, showing disintegrated inflammatory cells and proteinaceous debris. Despite strong initial clinical suspicion, a comprehensive TB workup, including acid-fast *Bacillus* (AFB) smears and TB polymerase chain reaction (PCR) from both sputum and pleural fluid, was persistently negative. Initial pleural fluid bacterial cultures were sterile (no aerobic or anaerobic growth).

Advanced imaging and hospital course

**Figure 2:**
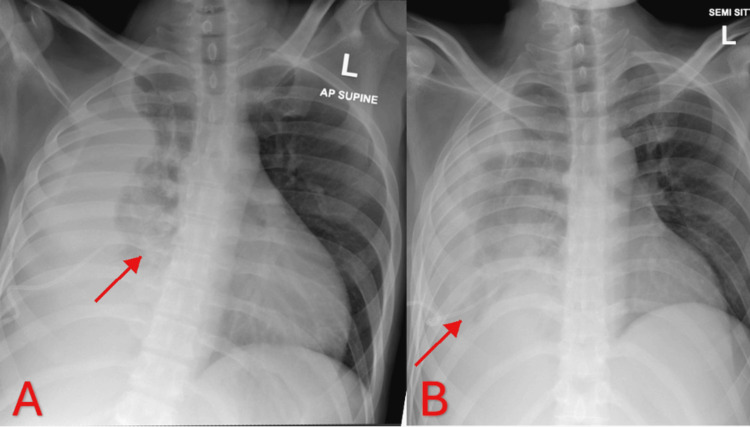
Serial chest radiographs demonstrating the clinical course (A) Anteroposterior (AP) supine chest radiograph on admission demonstrating complete opacification of the right hemithorax with contralateral mediastinal shift, consistent with a large right-sided pleural effusion. (B) Semi-sitting anteroposterior chest radiograph following chest tube insertion, showing partial re-expansion of the right lung with residual right lower zone opacity and a more central mediastinal position, indicating interval improvement.

The patient was initially started on ceftriaxone, metronidazole, and azithromycin (the latter was later discontinued upon exclusion of atypical pneumonia). A right intercostal drainage catheter was inserted, resulting in interval clearing of the pleural collection and partial re-expansion of the right lung on follow-up supine and semi-sitting radiographs, as shown in Figure 2.

**Figure 3:**
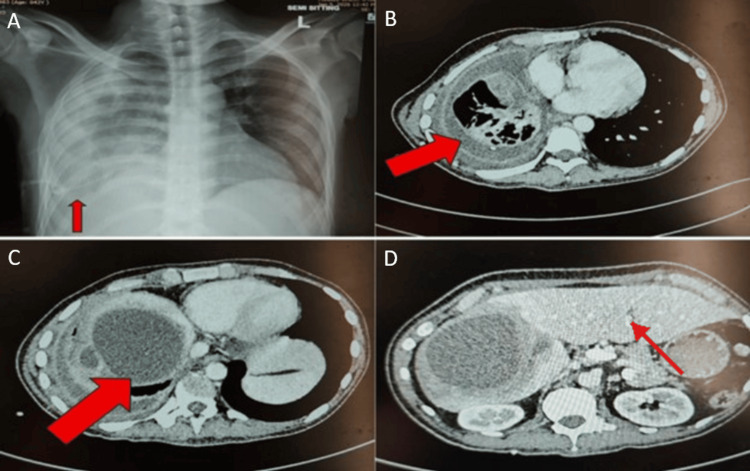
Radiological findings demonstrating pleural empyema secondary to complicated hepatic cysts (A) Semi-sitting anteroposterior chest radiograph showing right basal haziness with a right-sided intercostal chest drain in situ (arrow). (B) Contrast-enhanced axial CT of the chest demonstrating a right-sided heterogeneous pleural collection with internal air-fluid levels (arrow) and parenchymal involvement, consistent with empyema thoracis. (C) Contrast-enhanced axial CT at the level of the upper abdomen revealing a large complex multiloculated hepatic cyst with internal septations and a dependent fluid level in the right lobe of the liver (arrow). (D) Contrast-enhanced axial CT at the level of the mid-abdomen showing a right hepatic hypodense lesion without active enhancement, in keeping with a complicated simple hepatic cyst.

Contrast-enhanced CT of the chest and upper abdomen revealed a loculated right pleural collection with a split pleura sign, consistent with empyema. Unexpectedly, the abdominal CT cuts demonstrated marked hepatomegaly elevating the right hemidiaphragm, accompanied by multiple well-defined, multiloculated, and communicating hypodense cystic lesions with surrounding parenchymal edema, as shown in Figure 3. The largest lesion measured 12.5 x 9 cm and was located in the right hepatic lobe. Mass effect was noted, with compression of the intrahepatic inferior vena cava (IVC) and right branches of the portal vein. Notably, the lesion in segment VIII reached the peripheral subcapsular location, raising high suspicion of a disrupted hepatic capsule at the superior aspect with transdiaphragmatic rupture and decompression into the right pleural space.

Definitive diagnosis via liver aspiration

**Figure 4:**
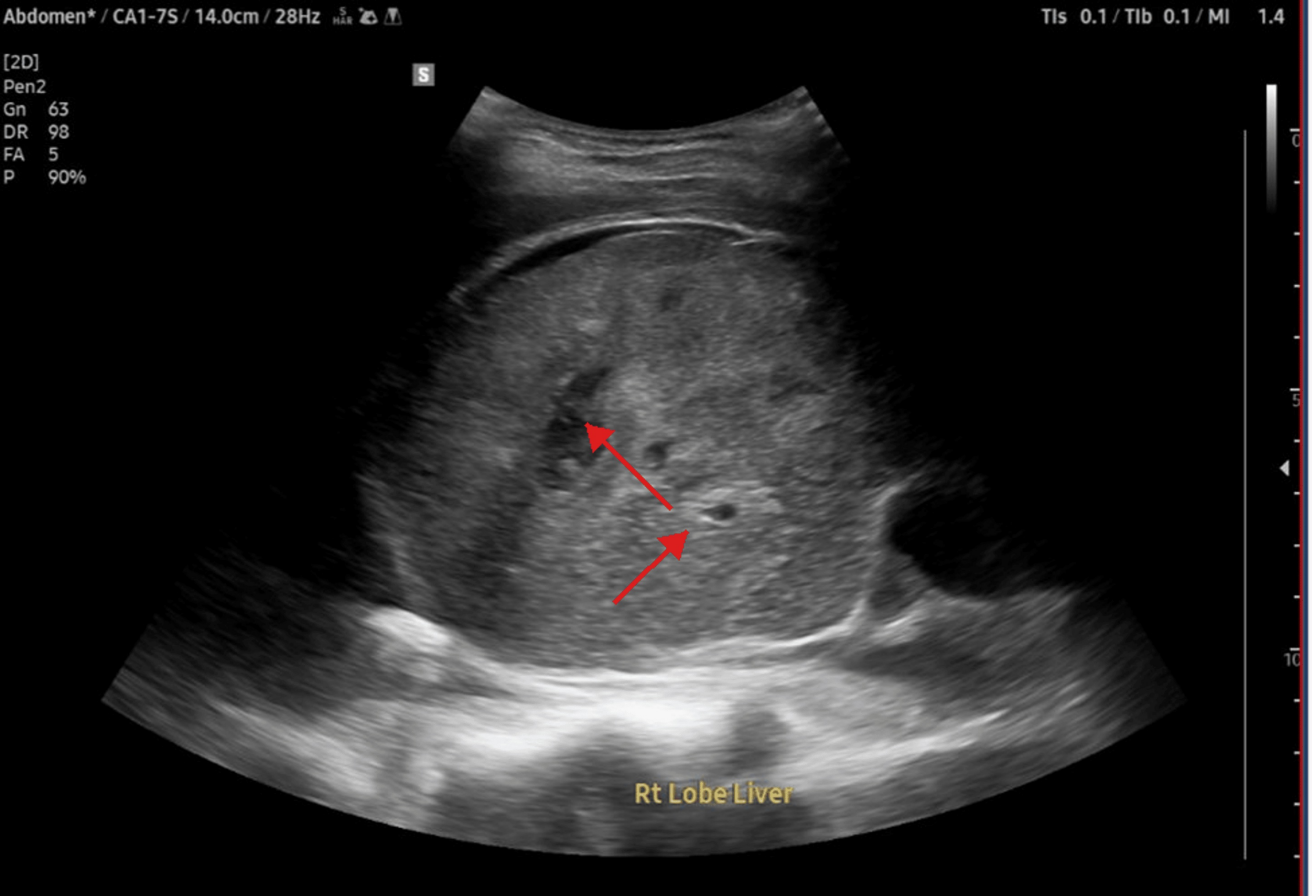
Initial abdominal ultrasound (prior to aspiration) demonstrating the large, well-defined 12.5 x 9 cm cystic lesion in the right lobe of the liver

Given the differential diagnosis of a pyogenic liver abscess (PLA) versus a complicated hydatid or amebic cyst, General Surgery and Infectious Disease consultations were obtained. Extensive serological and parasitological testing was performed: stool cultures were negative for *Salmonella*, *Shigella*, ova, and parasites, while serology for *Echinococcus* IgG and *Entamoeba histolytica* IgG returned negative. An ultrasound-guided percutaneous aspiration of the liver lesion was performed, as can be seen in Figure 4.

Macroscopic and microscopic evaluation of the aspirate revealed thick, sticky, purulent material with heavy (4+) white blood cells and Gram-negative rods on Gram stain. Fluid cultures definitively grew *Klebsiella pneumoniae*, confirming a diagnosis of PLA. The isolate was susceptible to amoxicillin-clavulanate, ceftriaxone, cefuroxime, ciprofloxacin, gentamicin, and piperacillin-tazobactam.

Follow-up and outcome

**Figure 5:**
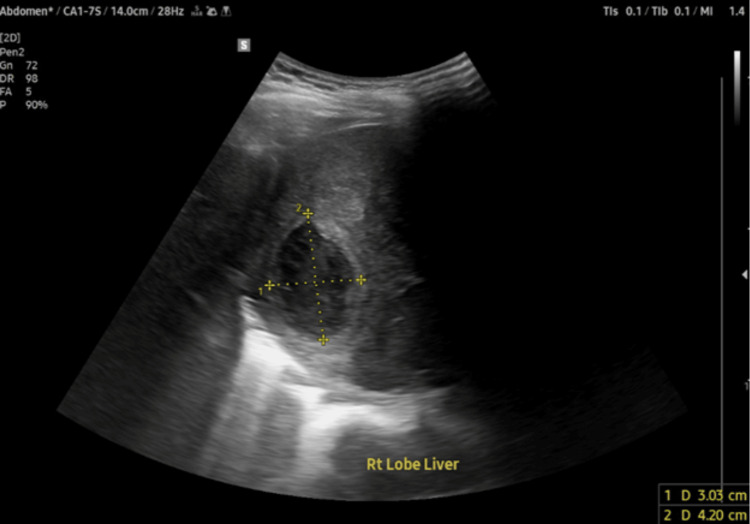
Follow-up abdominal ultrasound showing the right hepatic lobe drainage stent in situ and an interval regression in the size of the hepatic abscess, with measuring calipers indicating the residual lesion is 3.03 cm x 4.20 cm

Targeted antimicrobial therapy with ceftriaxone was continued for the disseminated *Klebsiella *syndrome for two weeks. A repeat abdominal ultrasound demonstrated the right hepatic lobe drainage stent in situ and an interval regression in both the size and number of the hepatic abscesses, with the largest residual lesion measuring 3.0 x 4.2 cm, as shown in Figure 5.

Thoracic surgery was consulted for the management of the organized right-sided empyema, and the patient was accepted for a surgical decortication. However, the patient refused the surgical procedure, opting instead to be discharged to follow up on his condition in his home country.

## Discussion

This case highlights a diagnostically challenging presentation of right-sided empyema thoracis initially managed as suspected pulmonary TB; however, the CT reported a possibility of a transdiaphragmatic extension from a *K. pneumoniae* PLA. In patients from TB-endemic regions, constitutional symptoms such as weight loss, night sweats, and massive unilateral pleural effusion frequently prompt presumptive TB isolation and treatment pathways [3,11]. However, this case emphasizes that aggressive *Klebsiella* infections must be actively considered as a differential diagnosis in high-risk demographic groups.

The pleural fluid profile in this patient, characterized by markedly elevated LDH, critically low glucose, and neutrophil predominance, was indicative of advanced bacterial empyema and strongly argued against tuberculous pleuritis, which typically demonstrates a lymphocytic predominance [1,3,4,12]. Negative TB molecular tests and AFB smears should immediately prompt clinicians to search for extra-pulmonary bacterial sources. In this patient, a cross-sectional CT, covering the upper abdomen, was pivotal in identifying the "silent" liver abscess [8]. PLAs can present with respiratory symptoms that entirely overshadow typical hepatic signs, such as right upper quadrant pain, leading to significant diagnostic delays.

*K. pneumoniae* is increasingly recognized as the dominant pathogen causing PLA, particularly in Asian populations [13]. The pathogen is notorious for its hypermucoviscosity (often associated with K1/K2 strains) and its capacity to cause an invasive, disseminated *Klebsiella *syndrome. The right lobe of the liver shares a close anatomical relationship with the diaphragm, particularly at the "bare area," which facilitates the direct lymphatic, hematogenous, or contiguous spread of infection into the pleural space [8]. The spontaneous rupture of a peripheral subcapsular hepatic lesion can lead to a hepatopleural fistula, causing direct purulent contamination of the thorax, as suspected in our patient [4,8].

From a regional perspective, PLA complicated by empyema is an important clinical entity in the Gulf. A recent Saudi Arabian case series [8] described multiple cases of PLA complicated by empyema, highlighting diverse underlying etiologies including *Klebsiella* infections. The high regional prevalence of diabetes mellitus, a major risk factor and poor prognostic indicator for invasive *Klebsiella* infections, may increase population susceptibility to these complex, cross-compartment infections.

The successful management of KPLA with transdiaphragmatic extension requires a combined therapeutic modality. Adequate source control through the simultaneous drainage of both the hepatic and pleural compartments is the cornerstone of therapy [8], supplemented by prolonged, targeted antimicrobial courses. This case underscores a critical take-home message for clinicians: always evaluate the subdiaphragmatic space in cases of unexplained right-sided empyema, and beware of KPLA as a formidable mimic of pulmonary TB.

## Conclusions

This case illustrates that constitutional symptoms and epidemiological risk factors, while indispensable in shaping the initial differential diagnosis, are insufficient alone to establish a definitive etiology in complex pleural disease. The clinical presentation, marked by night sweats, significant weight loss, and a history of travel to a high-prevalence region, created a compelling but ultimately misleading picture of tuberculous pleural effusion. The diagnosis of complicated bacterial empyema was confirmed only through the systematic integration of pleural fluid biochemistry, cytology, and advanced cross-sectional imaging. This highlights a fundamental principle in pulmonology: when clinical suspicion and objective data diverge, objective data must prevail. Clinicians managing massive pleural collections in endemic settings must remain vigilant against anchoring bias and should pursue a comprehensive, stepwise diagnostic workup. Timely pleural drainage, empirical antimicrobial therapy, and nutritional optimization are critical components of management in patients presenting with this degree of physiologic compromise. This case also reinforces the value of contrast-enhanced CT of the thorax as an early adjunct in characterizing complex pleural collections, guiding drainage strategy, and monitoring treatment response. Future prospective studies incorporating biomarker panels and point-of-care molecular diagnostics may further refine the differentiation of TB-mimicking empyema in resource-limited, high-burden settings.
